# Integrated Whole-Exome and Transcriptome Sequencing of Sporadic Parathyroid Adenoma

**DOI:** 10.3389/fendo.2021.631680

**Published:** 2021-05-14

**Authors:** Ya Hu, Xiang Zhang, Ou Wang, Ming Cui, Xiaobin Li, Mengyi Wang, Surong Hua, Quan Liao

**Affiliations:** ^1^ Department of General Surgery, Peking Union Medical College Hospital, Chinese Academy of Medical Sciences & Peking Union Medical College, Beijing, China; ^2^ Laboratory of Endocrinology, Department of Endocrinology, National Health Commission, Peking Union Medical College Hospital, Chinese Academy of Medical Sciences & Peking Union Medical College, Beijing, China

**Keywords:** parathyroid diseases, hyperparathyroidism, high-throughput, nucleotide sequencing, gene expression profiling

## Abstract

**Purpose:**

Hyperparathyroidism is the third most common endocrine disease. Parathyroid adenoma (PA) accounts for approximately 85% of cases of primary hyperparathyroidism, but the molecular mechanism is not fully understood. Herein, we aimed to investigate the genetic and transcriptomic profiles of sporadic PA.

**Methods:**

Whole-exome sequencing (WES) and transcriptome sequencing (RNA-seq) of 41 patients with PA and RNA-seq of 5 normal parathyroid tissues were performed. Gene mutations and characterized expression changes were identified. To elucidate the molecular mechanism underlying PA, unsupervised consensus clustering of RNA-seq data was performed. The correlations between the sequencing data and clinicopathological features of these patients were analyzed.

**Results:**

Previously reported PA driver gene mutations, such as *MEN1* (9/41), *mTOR* (4/41), *ZFX* (3/41), *CASR* (3/41), *EZH2* (2/41) and *FAT1* (2/41), were also identified in our cohort. Furthermore, somatic mutation of *EZH1*, which had not been reported in PA, was found in 4 samples. RNA-seq showed that the expression levels of 84 genes were upregulated and 646 were downregulated in PA samples compared with normal samples. Unsupervised clustering analysis of RNA-seq data clustered these patients into 10 subgroups related to mutation or abnormal expression of a group of potential pathogenic genes.

**Conclusion:**

*MEN1*, *EZH2*, *CASR*, *EZH1, ZFX*, *mTOR* and *FAT1* mutations in PA were revealed. According to the RNA-seq data clustering analysis, cyclin D1, β-catenin, VDR, CASR and GCM2 may be important factors contributing to the PA gene expression profile.

## Introduction

Primary hyperparathyroidism (pHPT) is a common endocrine disorder with an incidence of approximately 66 and 25 per 100000 person-years among women and men, respectively (1). It was reported that the prevalence of pHPT was as high as 2.1% in postmenopausal women (2). The parathyroid tumor tissue secretes excessive parathyroid hormone, which results in hypercalcemia and related complications, such as osteoporosis, bone fracture, urolithiasis and renal failure. Parathyroid adenoma (PA) accounts for approximately 85% of pHPT. The genetic mechanisms of PA are not fully known, even though tremendous efforts have been made in previous studies. In PA with a hereditary background, the most well-known driver gene of PA was multiple endocrine neoplasia type 1 (*MEN1*). Other possible driver genes for hereditary pHPT include *RET* in MEN-2, *CDKN1B* in MEN-4, *CDC73* in hyperparathyroidism-jaw tumor syndrome, *GCM2* in familial isolated pHPT, *CASR* in familial hypocalciuric hypercalcemia type 1, *GNA11* in familial hypocalciuric hypercalcemia type 2, and *AP2S1* in familial hypocalciuric hypercalcemia type 3. For sporadic PA, somatic mutation of *MEN1* was identified in approximately 35% of cases (3). Mutation of *CCND1* or overexpression of cyclin D1 was found in 30% of sporadic PA cases. Other genes, such as *CASR*, *EZH2*, *CDKI*, and *CTNNB1*, may also contribute to the tumorigenesis of PA. However, the driver genes could still not be identified in a substantial proportion of sporadic PA.

Several studies have explored the molecular mechanism of PA with next-generation sequencing (NGS) technologies, such as whole-exome sequencing (WES) or transcriptome sequencing (RNA-seq). In 2018, Wei et al. (4) used WES to explore genetic abnormalities in 20 specimens of PAs. In 2019, Chai et al. (5) performed RNA-seq to compare the differentially expressed genes (DEGs) between 10 PA and 5 normal parathyroid (PaN) samples, and 8 hub molecules, including RPL23, RPL26, RPN1, RPS25, SEC11A, SEC11C, SEC61G and SPCS2, were identified. All these previous studies focused on one platform for tumor genome profiling. However, a multiplatform NGS analysis combined with WES and RNA-seq provides a more comprehensive view of the genetic landscape. RNA-seq is adept for identifying gene expression changes, gene fusions and alternative splicing, while WES is good at detecting copy number variants and gene mutations with low expression levels. With the corresponding RNA-seq data, the functional effects of genetic variants from WES can be interpreted with confidence.

Here, we performed concomitant WES and RNA-seq in 41 PA samples to identify related genetic variants and the consequent transcriptomic changes. The somatic mutations and DEGs were identified, and integrated analysis was employed. Based on RNA-seq data, unsupervised clustering with consensus clustering was applied to classify the patients, and the possible underlying molecular mechanism was explored.

## Materials and Methods

### Patients and Specimens

A total of 41 samples of PA were selected randomly from the tissue bank of parathyroid tumors in the present study. These patients received operations from 2013 to 2019 at Peking Union Medical College Hospital, a tertiary referral university hospital. All the patients had a single PA without a history of multiple endocrine neoplasia, familial hyperparathyroidism syndrome or neck irradiation. The diagnosis of PA was made pathologically according to the WHO 2017 criteria (6). During the operation, the tumor specimens were collected from the central portion of the tumors to avoid contamination from adjacent tissues. Then, the specimens were immediately immersed in RNAlater (Ambion Inc., USA) solution at 4°C and stored at -80°C until use. The peripheral blood samples were collected, and white blood cells (WBCs) were stored at -80°C. Tiny PaN tissues were incidentally obtained from 5 patients with normal parathyroid function during surgery for thyroid diseases. The clinical data, including age, sex, imaging results, biochemical results and pathological evaluation, were collected. Follow-up data was obtained by reviewing outpatient records and telephone interviews. This study was approved by the Institutional Ethics Review Board of Peking Union Medical College Hospital (S-K836).The written informed consent was obtained from all the participants.

### WES and WES Data Analysis

Genomic DNA was extracted from tumor and WBC samples with a DNeasy Blood & Tissue Kit (QIAGEN, Germany). A total of 0.6 μg of DNA per sample was used to prepare the DNA libraries with the Agilent SureSelect Human All Exon V6 Kit (Agilent Technologies, CA, USA). Then, the DNA libraries of each sample were sequenced with the Illumina HiSeq platform, and 150-bp paired-end reads were produced. Low-quality reads and reads containing adapter contamination were filtered. With Burrows-Wheeler Aligner (BWA) software, valid sequencing reads were mapped to the reference human genome (B37). Samtools, ANNOVAR, 1000 Genomes and other related databases were used to identify and annotate SNPs and insertions and deletions (InDels). The somatic single-nucleotide variant (SNV) was detected by MuTect, and the somatic InDel was detected by Strelka (7, 8). Somatic variants in the segmental duplication or with a frequency > 0.01 in the 1000 Genomes Chinese database were excluded for further analysis. The copy number variant was identified with Control-FREEC (9). The significantly mutated genes (SMGs) were identified by MuSiC software (10).

### Transcriptome Sequencing and RNA-seq Data Analysis

RNA was extracted from tissue samples with TRIzol™ Reagent (Invitrogen, USA). A total of 2 μg of RNA per sample was used to generate sequencing libraries with the NEBNext^®^ UltraTM RNA Library Prep Kit for Illumina^®^ (NEB, USA). RNA-seq was completed on an Illumina HiSeq platform, and 150-bp paired-end reads were produced. Paired-end clean reads were aligned to the reference genome using HISAT2 v2.0.5. HTSeq was used to count the reads mapped to each gene, and the fragments per kilobase of transcript sequence per million base pairs sequenced (FPKM) value was calculated based on the length of the gene and the read count mapped for each gene. Gene set enrichment analysis (GSEA) was used for functional enrichment analysis of DEGs, including Gene Ontology (GO) and Kyoto Encyclopedia of Genes and Genomes (KEGG) analyses (11). Gene fusion was identified by STAR-Fusion (12). The expression network analysis based on the DEGs was performed using the OmicStudio tools (https://www.omicstudio.cn/tool). For each pair of analyzed DEGs, the Pearson correlation coefficient was greater than 0.95 (p<0.001).

### Integrative WES and RNA-seq Analysis

To explore the possible molecular mechanism underlying PA, unsupervised clustering of RNA-seq data was performed with the R package ConsensusClusterPlus. Consensus clustering was used to aid in discovering the optimal class. The number of clusters was selected according to the delta area plot and consensus cumulative distribution function (CDF) plot. Furthermore, the clustering results were explored with the features of the expression profile and genes in each cluster. The flowchart of data analysis was shown in **Figure S1**.

### Statistical Analysis

Continuous parameters are shown as the mean ± standard deviation (SD), and discrete data are reported as numbers or corresponding percentages. Differences in variables between groups were evaluated with the Mann-Whitney U test. Fisher’s exact test was used to compare the categorical data. A two-sided p-value < 0.05 was set as the threshold for statistical significance. All statistical analyses were performed with SPSS version 16.0.

## Results

### Clinical Characteristics of Patients With PA

**Figure 1:**
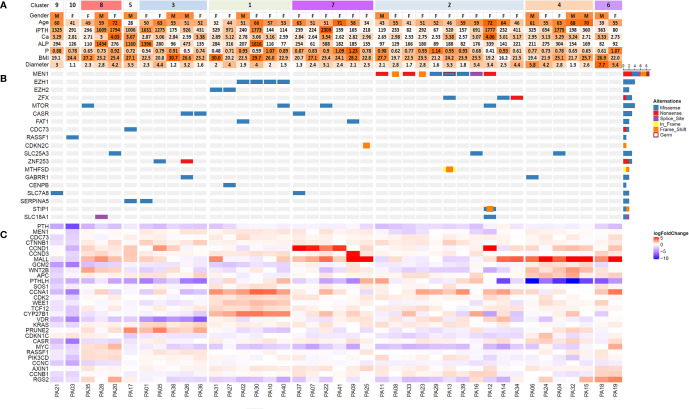
Heatmap of clinical features **(A)**, significantly mutated genes **(B)** and differentially expressed genes **(C)** among 41 patients with parathyroid adenoma. Samples were clustered based on RNA-seq data by unsupervised clustering with the ConsensusClusterPlus package. Sample numbers are shown below the corresponding columns. In part **(C)** the logarithm of fold change in gene expression compared with the expression levels in normal parathyroid tissue is visualized by color intensity. Upregulation and downregulation of gene expression are represented by red and blue, respectively. iPTH, serum intact PTH level (12-67 pg/ml); Ca, serum calcium level (2.13-2.70 mmol/L); P, serum phosphorus level (0.81-1.45 mmol/L); ALP, alkaline phosphatase (35-100 U/L); BMI, body mass index; Diameter, maximum diameter of the tumor (cm).

A total of 41 patients were enrolled in the present study (**Figure 1A**). The average age at diagnosis was 53.9 ± 10.8 (28–72) years. An obvious female predominance was revealed, with a female-to-male ratio of 30:11. The average serum levels of iPTH and Ca were 633.7 ± 628.1 (67.1-2309.2) pg/ml and 3.01 ± 0.37 (2.53-4.06) mmol/L, respectively.

### Gene Mutations and Copy Number Variations With WES

For WES, we achieved a mean sequencing depth of 294× and 140× for the tumor and WBC control, respectively. Thereinto, 91.4% of the exome in tumors and 80.1% of the exome in WBC controls was sequenced with a depth of >50×. According to our filter criteria, a total of 668 nonsynonymous somatic mutations were detected, which included 636 SNVs and 32 InDels (**Table S1**). The top 7 recurrent mutated genes for PA were *MEN1* (9/41), *EZH1* (4/41), *mTOR* (4/41), *ZFX* (3/41), *CASR* (3/41), *EZH2* (2/41) and *FAT1* (2/41) (**Figure 1B**, **Table 1**). In this cohort, 8 somatic mutations and 1 germline mutation of *MEN1* were identified in 9 PA samples. Other potential driver genes reported in COSMIC, such as *RASSF1*, *CDKN2C* and *CDC73*, were found in one sample.

**Table 1:** Recurrently mutated genes and potential driver genes in parathyroid adenomas.

Gene	Sample ID	Somatic/Germline	Mutation type	Amino acid change
MEN1	8	Somatic	frameshift deletion	NM_000244: exon2:c.269_270del:p.Y90fs
MEN1	16	Somatic	splicing	NM_000244: exon8:c.928-1G>T
MEN1	23	Somatic	frameshift insertion	NM_000244: exon2:c.309dupG:p.S104fs
MEN1	29	Somatic	missense SNV	NM_000244: exon2:c.C95G:p.P32R
MEN1	33	Somatic	stopgain	NM_000244: exon3:c.C511T:p.Q171X
MEN1	39	Somatic	missense SNV	NM_000244: exon9:c.T1282A:p.W428R
MEN1	11	Somatic	stopgain	NM_000244: exon9:c.C1339T:p.Q447X
MEN1	12	Somatic	stopgain	NM_000244: exon4:c.C787T
MEN1	13	Germline	missense SNV	NM_000244: exon2:c.A1G:p.M1V
EZH1	2	Somatic	missense SNV	NM_001991: exon17:c.A1925T:p.Y642F
EZH1	10	Somatic	missense SNV	NM_001991: exon17:c.A1925T:p.Y642F
EZH1	30	Somatic	missense SNV	NM_001991: exon17:c.A1925T:p.Y642F
EZH1	40	Somatic	missense SNV	NM_001991: exon17:c.A1925T:p.Y642F
MTOR	22	Somatic	missense SNV	NM_004958: exon53:c.A7257T:p.E2419D
MTOR	35	Somatic	missense SNV	NM_004958: exon47:c.C6644T:p.S2215F
MTOR	40	Somatic	missense SNV	NM_004958: exon27:c.T4079C:p.L1360S
MTOR	12	Somatic	missense SNV	NM_004958: exon56:c.A7501T:p.I2501F
ZFX	13	Somatic	missense SNV	NM_001178086: exon4:c.A371G:p.K124R
ZFX	34	Somatic	stopgain	NM_001178086: exon6:c.G658T:p.E220X
ZFX	14	Somatic	missense SNV	NM_001178086: exon6:c.C1603T:p.R535W
CASR	26	Somatic	missense SNV	NM_000388: exon3:c.A307C:p.T103P
CASR	37	Somatic	missense SNV	NM_000388: exon3:c.C413T:p.T138M
CASR	36	Somatic	missense SNV	NM_000388: exon7:c.T1753C:p.C585R
EZH2	31	Somatic	missense SNV	NM_001203249: exon15:c.T1768A:p.Y590N
EZH2	27	Somatic	missense SNV	NM_001203249: exon15:c.A1769T:p.Y590F
FAT1	2	Somatic	missense SNV	NM_005245: exon10:c.G8140C:p.E2714Q
FAT1	9	Somatic	missense SNV	NM_005245: exon10:c.G8671T:p.D2891Y
CDC73	17	Somatic	missense SNV	NM_024529: exon3:c.G268T:p.D90Y
CDC73	17	Somatic	stopgain	NM_024529: exon1:c.G128A:p.W43X
RASSF1	3	Somatic	missense SNV	NM_170713: exon2:c.G157T:p.V53L
CDKN2C	25	Somatic	frameshift deletion	NM_078626: exon2:c.397_413del:p.R133fs

### DEGs and Unsupervised Clustering With RNA-seq Data

**Figure 2:**
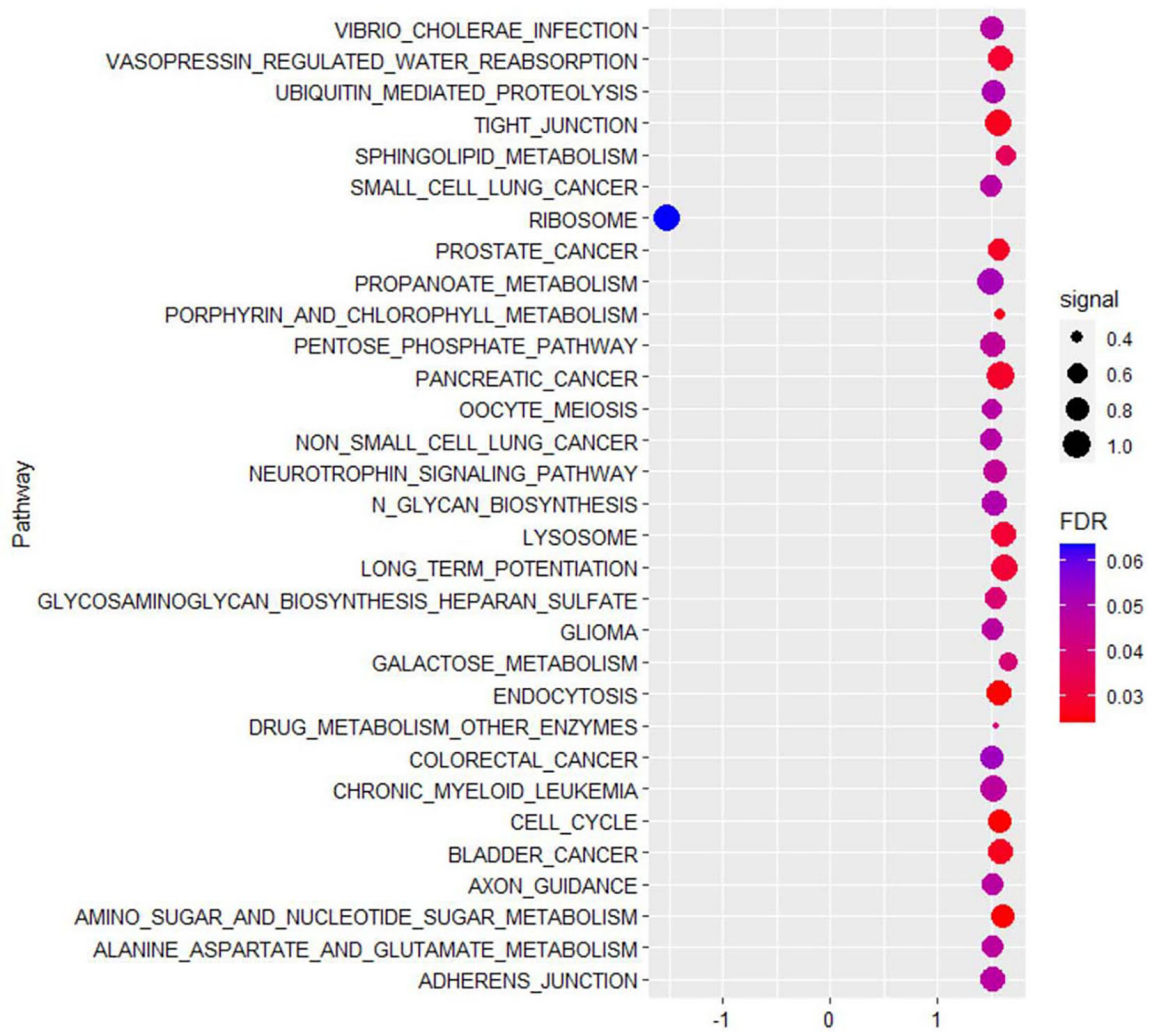
The top 30 KEGG pathways enriched by gene set enrichment analysis (GSEA) showed that differentially expressed genes of parathyroid adenoma were enriched in multiple biological processes, such as ribosome, lysosome, cell cycle and tight junction. The horizontal axis represents the normalized enrichment score (NES), and the vertical axis represents the KEGG pathways. The false discovery rate (FDR) value and enrichment signal are represented by the color and circle diameter, respectively.

In 41 PA and 5 PaN samples, a mean of 55.7 million reads were obtained by RNA-seq per sample, and 96.3% reads were successfully aligned to the reference genome. Compared with PaN samples, 84 genes were upregulated and 646 genes were downregulated (with a fold change of 1.5) in PA samples. GSEA showed that the DEGs were enriched in multiple biological processes, such as ribosome, lysosome, cell cycle and tight junction (**Figure 2**). Then, the DEGs were selected to build the expression network. The network comprised 113 network nodes and 500 network edges, indicating a complicated regulatory association in PA (**Figure S2**).

### Integrative Analysis of WES and RNA-seq Data

**Figure 3:**
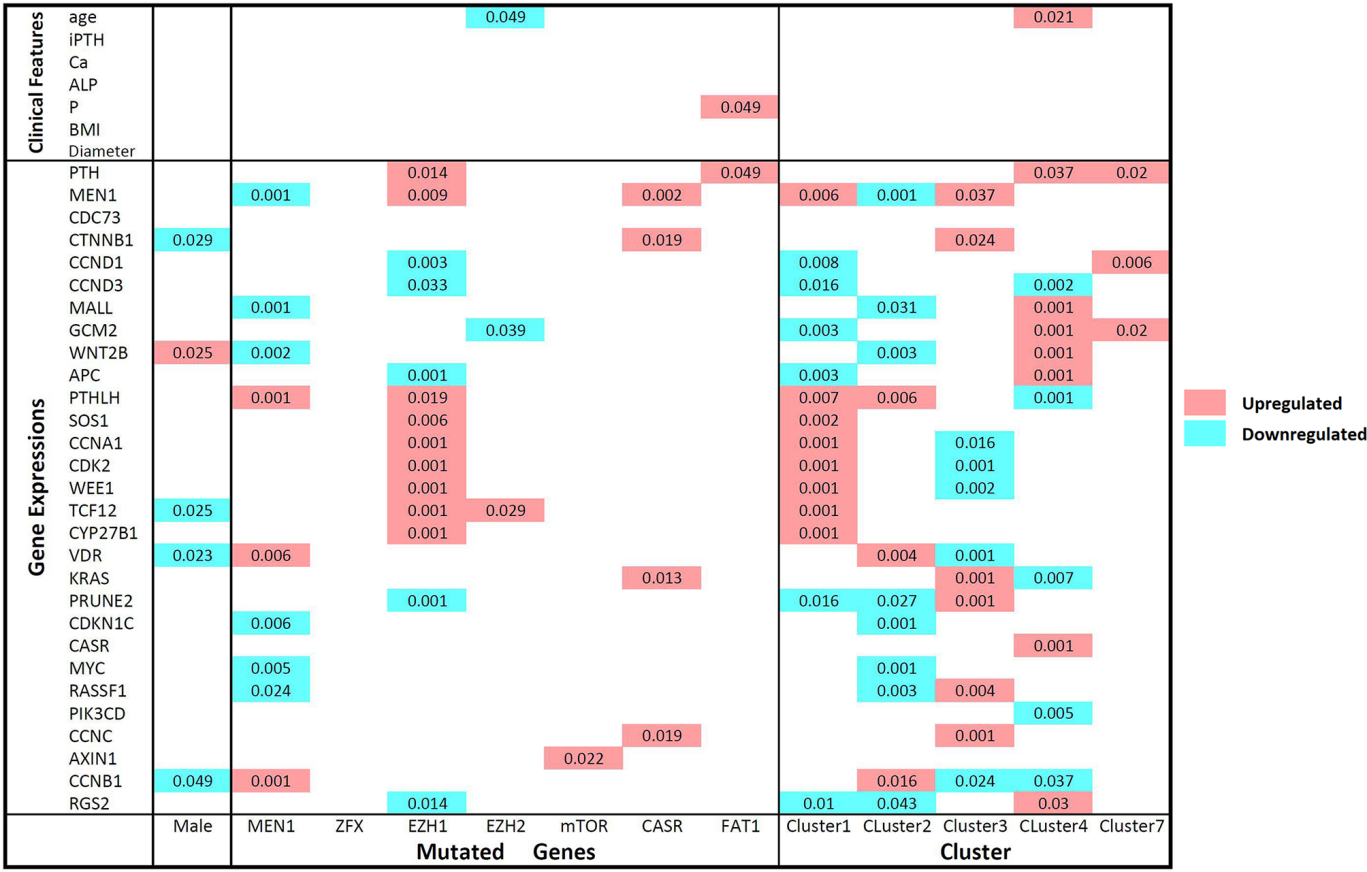
Comparisons of gene expression levels and clinical features between mutant and wild-type parathyroid adenomas and between clusters according to the Mann-Whitney U test. The names of the mutated genes and clusters names are listed on the horizontal axis, and the gene expression level and clinical features are shown on the vertical axis. Comparisons of wild-type and mutant parathyroid adenomas and different clusters: upregulated and downregulated expression levels in samples with the mutant genotype or in certain clusters are represented by red and blue, respectively. P-values less than 0.05 are marked in the matrix. iPTH, serum intact parathyroid hormone level; Ca, serum calcium level; ALP, serum alkaline phosphatase level; P, serum phosphorus level; BMI, body mass index; Diameter, maximum diameter of the parathyroid adenoma.

Through consensus clustering, the RNA-seq data of PA samples could optimally be categorized into 10 clusters (**Figure S3**). Although unsupervised clustering is based merely on a mathematical calculation of gene expression data, prominent molecular features could be identified for most clusters based on existing knowledge (**Figure 1C**). On one hand, previously reported driver gene mutations could be identified in 3 clusters (Clusters 1, 2 and 5). In Cluster 2, all 9 samples with *MEN1* mutations (8 somatic mutations and 1 germline mutation) and 2 samples with *ZFX* mutations were included, which indicated that *MEN1* and *ZFX* mutations may result in a specific and similar expression profile. The expression of MEN1 in samples with *MEN1* mutations was decreased significantly (p<0.001). In Cluster 1, mutations in *EZH2* and *EZH1* were identified, and the expression levels of SOS1 (p= 0.002), CCNA1 (p < 0.001), CDK2 (p< 0.001), WEE1 (p< 0.001), TCF12 (p< 0.001) and CYP27B1 (p< 0.001) were significantly upregulated compared with those in the other subgroups (**Figure 3**). Double somatic mutations of *CDC73*, a stop-gain mutation (NM_024529:exon1:c.G128A:p.W43X) and a missense mutation (NM_024529:exon3:c.G268T:p.D90Y), were identified in PA17 (Cluster 5). Three samples with somatic mutations of *CASR* were distributed in Cluster 3 and Cluster 7.

On the other hand, prominent gene expression abnormalities were found in some clusters as previously reported in PA. The cyclin D1 (CCND1) mRNA level in Cluster 7 was much higher than that in any other cluster (p=0.006). For GCM2 and WNT2B, all 5 samples with the highest expression levels were clustered into Cluster 4, while no genetic mutation of known driver genes was identified. High expression levels of AXIN1 and CCNB1 were found in Cluster 6. High levels of KRAS (p=0.001) and PRUNE2 (p=0.001), as well as low levels of vitamin D receptor (VDR) (p=0.001), were prominent in Cluster 3. Low levels of CASR and high levels of MYC were found in the three samples in Cluster 8. Sample PA21 in Cluster 9 and sample PA03 in Cluster 10 had low levels of CDC73, CASR and SOS1, while no copy number or gene fusion abnormality of these genes was found.

### Relationship Between Gene Expression and Clinical Features

**Figure 4:**
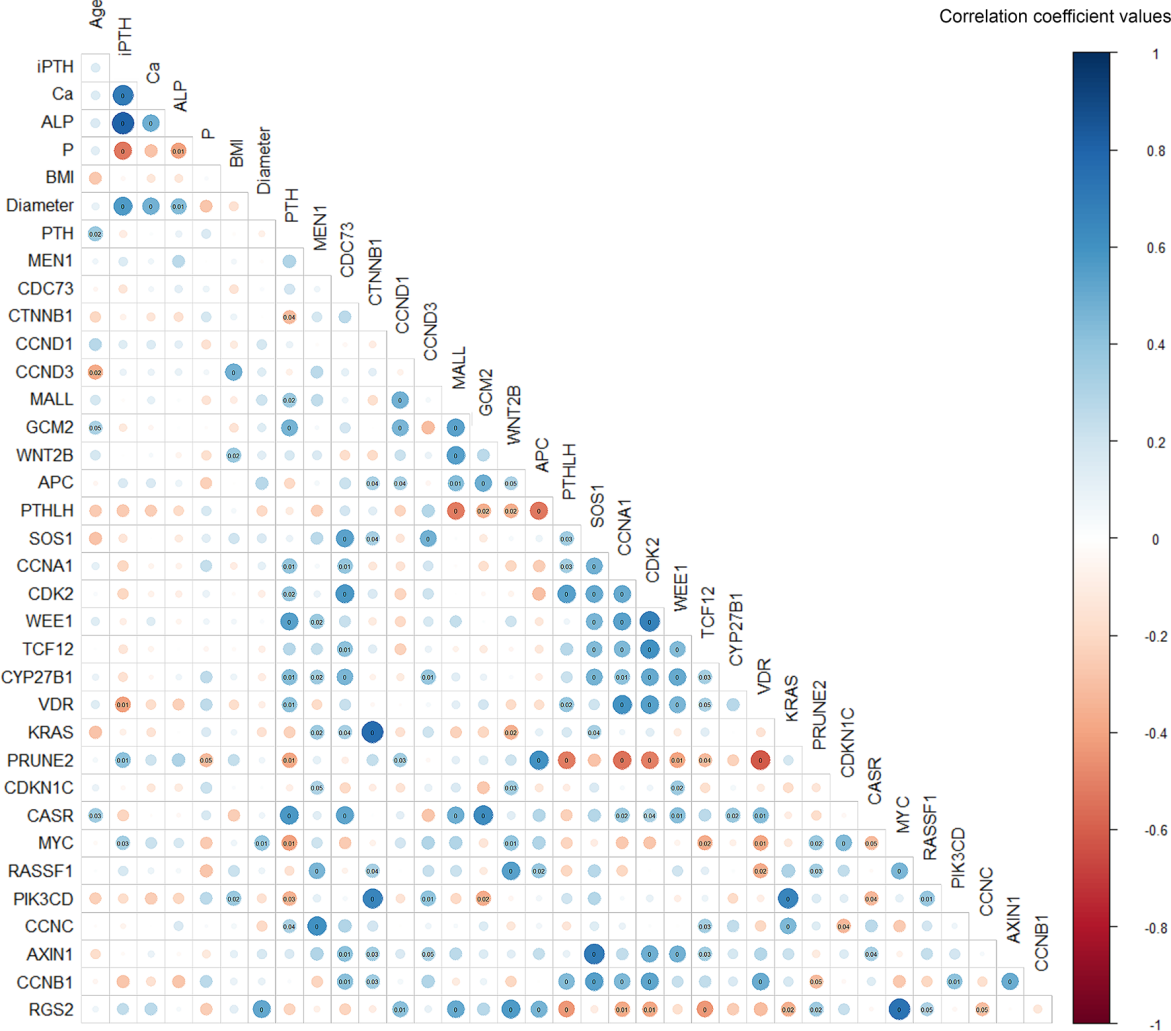
Spearman’s correlation matrix between gene expression and clinicopathological features in parathyroid adenoma. The correlation coefficient values are visualized by the size and color intensity of the circles. Positive and negative correlations are represented by blue and red, respectively. P-values below 0.05 are marked in the matrix, with P-values below 0.01 marked as zero. iPTH, serum intact parathyroid hormone level; Ca, serum calcium level; ALP, serum alkaline phosphatase; P, serum phosphorus level; BMI, body mass index; Diameter, maximum diameter of the parathyroid adenoma.

The serum level of iPTH was correlated with serum Ca (p<0.001, rs=0.685), alkaline phosphatase (ALP) (p<0.001, rs=0.802), phosphorus (P) (p<0.001, rs=-0.525) and tumor diameter (p<0.001, rs=0.570), as well as the expression levels of VDR (p=0.009, rs=-0.403) and PRUNE2 (p=0.009, rs=0.401) (**Figure 4**). The expression level of the *PTH* gene was found to be correlated with the expression of multiple genes, such as *CTNNB1* (p=0.045, rs=-0.315), *MALL* (p=0.024, rs=0.352), and *GCM2* (p=0.002, rs=0.468).

## Discussion

To our knowledge, this study is the first integrative analysis of WES and RNA-seq in parathyroid neoplasia, providing new insight into the molecular mechanism of PA. Using WES, we revealed a group of mutations in genes such as *MEN1*, *EZH2*, *CDC73*, *ZFX*, *CASR*, *FAT1* and *RASSF1*, almost all of which had been reported previously. In the RNA-seq analysis, overexpression of CCND1 and CTNNB1, which are recognized as vital factors for PA tumorigenesis, was also found (13, 14). KEGG analysis also found that ribosomal protein dysregulation was related to PA and is a known factor involved in tumor development (15). Previous work focused mainly on the mutation or expression of one or several of these related genes, which may be only one facet of the complex molecular mechanisms of PA tumorigenesis. However, contradictory results were occasionally reported in different publications. Therefore, we performed unsupervised clustering for the RNA-seq data, and 10 clusters were identified. Even though it is not easy to explain the intrinsic molecular mechanism underpinning these clusters from a single study with limited samples, we revealed some intriguing aspects.

In our cohort, 8 somatic mutations and 1 germline mutation of *MEN1* were identified, and the expression levels of *MEN1* in these 9 samples were significantly decreased. All these samples were clustered in one subgroup, indicating that the *MEN1* genotype might be related to the expression profile. *MEN1*, a tumor suppressor gene, was identified as a genetic driver of multiple endocrine neoplasia type 1 (16). Biallelic inactivation of *MEN1* is also found in approximately 12-35% sporadic PA cases (17, 18). We found that one patient with seemingly sporadic PA had a germline *MEN1* mutation, which was also reported by other authors (19). Three samples (7.3%) harboring somatic *ZFX* mutations were also included in Cluster 2, one of which carried a *MEN1* mutation concurrently. *ZFX*, a transcriptional target of cyclin D, was identified as a candidate driver gene for PA with a mutation rate of 5% (20, 21). In contrast to previous reports, all three mutations of *ZFX* in our cohort were not located at the R767 or R768 position.

Interestingly, 2 patients with the rare activating mutation Y464 of *EZH2* and 4 patients with the somatic mutation Y642F of *EZH1* were classified into one cluster (Cluster 1). Our results are consistent with previous reports showing that *EZH2* mutations are potential genetic drivers of PA. The activating mutation Y464N (previously described as Y641N) in *EZH2* was found previously in 2 of 193 sporadic PA samples (22). Although rare, 2 patients harbored the Y464 mutation in our cohort of 41. Furthermore, the somatic mutation Y642F of *EZH1* was identified in another 4 patients. Like *EZH2*, *EZH1* is also a component of the noncanonical polycomb repressive complex-2, which is recurrently mutated in multiple hematological malignancies (23). *EZH1* mutations were found in 20% of Hürthle cell adenomas and 10% of Hürthle cell carcinomas, and 53.8% of the mutations were Y642F (24). Though not reported previously, *EZH1* may be a potentially important gene for PA.

A somatic *CDC73* mutation was identified in one sample (PA17). Somatic inactivating mutations of *CDC73*, or loss of staining of the encoded parafibromin, occurred in approximately 60% of parathyroid carcinomas and in a few PAs (25, 26). In addition, significantly low expression of CDC73 was found in PA03 and PA21, while no mutation or copy number change was found in *CDC73*. Epigenetic changes may be the underlying cause.

In our cohort, overexpression of CCND1 was found in some samples, and the 4 samples with the highest expression levels were clustered in one group (Cluster 7). *CCND1*, encoding cyclin D1, was first identified as an oncogene in parathyroid tumors, and overexpression of this gene was found in 20-40% of PA samples (17, 27). Overexpression of CCND1 was caused by rearrangement of the PTH 5’ regulatory region of the *CCND1* coding region in some PAs. However, this could not be identified by WES in the present study. Neither gene amplification nor mutation of *CCND1* was found in these samples. Furthermore, another cell cycle regulator factor, cyclin D3 (*CCND3*), may also be involved in sporadic PA, as we found that the expression level of cyclin D3 in PA09 was approximately 31 times higher than that in the normal control. This may be caused by the copy gain of *CCND3* identified in this sample. The partners of cyclin D1 in cell cycle regulation, such as *CDKN1B*, *CDKN1C*, *CDKN2C* and other CDKI genes, were also reported to be involved in sporadic PA (28, 29). As reported, the expression of CDKN1B and CDKN1C was downregulated in some of our samples, while no somatic or germline variant of this gene was found (30). In addition, a frameshift deletion of *CDKN2C* was identified in one sample, and this mutation had been reported previously in a few PA samples (29, 31).

In the present cohort, somatic *CASR* mutations were identified in 3 samples, but the gene expression levels were not different from those in PaN. These 3 samples were clustered into different subgroups. *CASR* encodes a calcium sensing receptor, by which parathyroid cells monitor the extracellular calcium level. Inactivating germline mutations of *CASR* are responsible for familial hypocalciuric hypercalcemia and neonatal severe hyperparathyroidism. However, somatic inactivating *CASR* mutation was rarely found in sporadic PA (32–34). Therefore, *CASR* mutation was believed to be a predisposing factor rather than a genetic driver in PA (17). Another 3 specimens with low CASR expression were clustered into an independent subgroup (Cluster 8). Aberrant expression of *CASR* is frequently found in parathyroid tumors (35, 36). Five samples with the lowest VDR levels were clustered in Cluster 3, and VDR levels were negatively correlated with serum PTH levels, suggesting a vital role in PA. Decreased VDR expression was related to the high proliferation of pHPT, but no *VDR* mutation was found in our cohort (35, 37).

We could not identify any somatic or germline variants of *GCM2* in this Chinese cohort. However, a prominent increase in GCM2 levels was identified in 5 samples in an independent subgroup (Cluster 4). This was consistent with previous findings in which the GCM2 expression level was significantly upregulated in PA and correlated with a decrease in the response to hypocalcemia (38). *GCM2* encodes a transcription factor that is a critical regulator of the development of the parathyroid gland, and its activating germline variants are responsible for familial isolated hyperparathyroidism (39). In addition, Cluster 4 and 6 revealed no apparent driver mutation but showed aberrant expression of some tumor-related genes, such as GCM2 and AXIN1, indicating that driver gene plays an important, but not indispensable, role in the initiation of PA.

Another interesting mutation might be *SLC25A3*, which was identified in 3 of 41 samples. *SLC25A3* is essential for cytochrome c oxidase of the mitochondrial respiratory chain and *SLC25A3* deletion causes mitochondrial cardiomyopathy (40). Previously, mitochondrial variations may result in oncocytic phenotype of PA (41). However, *SLC25A3*-related studies were not available in PA.

There were several limitations to this study. The main shortcoming was the limited number of participants in this cohort, even though it was quite large considering recent sequencing studies on PA. Additional samples may help validate the gene mutation with low incidence. Second, due to the lack of normal parathyroid specimens for comparison, immunohistochemical staining or Western blot analysis were not suitable to confirm the protein levels of the related gene variants among PA samples. Third, bulk RNA-Seq data are heavily influenced by tissue cellular composition. The gene expression profile might contain not only the tumor-induced molecular alterations but also the interference from tumor heterogeneity.

## Conclusions

This study revealed almost all previously reported molecular features of PA using integrated WES and RNA-seq analyses. In addition to the previously reported mutant genes, such as *MEN1*, *EZH2*, *CASR* and *CDC73*, somatic mutations of *EZH1* were also identified in the present PA cohort. With clustering based on RNA-seq, abnormal expression of cyclin D1, β-catenin, VDR, CASR and GCM2 may be an important factor influencing the gene expression profile of PA.

## Data Availability Statement

The data presented in the study are deposited in the Genome Sequence Archive (GSA) in BIG Data Center, Beijing Institute of Genomics (BIG), Chinese Academy of Sciences (CAS), publicly accessible at https://bigd.big.ac.cn/gsa-human/, accession number (HRA000665).

## Ethics Statement

The studies involving human participants were reviewed and approved by the Institutional Ethics Review Board of Peking Union Medical College Hospital. The patients/participants provided their written informed consent to participate in this study.

## Author Contributions

YH and XZ designed the study and performed the experiments. YH, XL, and QL performed the surgery. XZ, OW, MC, XL, MW, and SH enrolled the patients and collected the samples. YH, XZ, OW and MC analyzed the data. YH, XZ and QL wrote and modified the manuscript. All authors contributed to the article and approved the submitted version.

## Funding

This work was supported by the Chinese Academy of Medical Sciences (CAMS) Innovation Fund for Medical Sciences (CIFMS) (2017-I2M-1-001), the Nonprofit Central Research Institute Fund of the Chinese Academy of Medical Sciences (2018PT32014) and the Peking Union Medical College Innovative Team Development Program.

## Conflict of Interest

The authors declare that the research was conducted in the absence of any commercial or financial relationships that could be construed as a potential conflict of interest.
