# Supplementary material for: Integrated Whole-Exome and Transcriptome Sequencing of Sporadic Parathyroid Adenoma
Source: Front Endocrinol (Lausanne). 2021 May 14;12:631680. doi: 10.3389/fendo.2021.631680 (PMC8163014; doi:10.3389/fendo.2021.631680)
Supplement: Supplementary file 1 [file DataSheet_1.docx]

Integrated Whole-Exome and Transcriptome Sequencing of Sporadic Parathyroid Adenoma

**Supplementary Figures**

Ya Hu^1#^, Xiang Zhang^1#^, Ou Wang^2^, Ming Cui^1^, Xiaobin Li^1^, Mengyi Wang^1^, Surong Hua^1^, Quan Liao^1*^

^1^Department of General Surgery, Peking Union Medical College Hospital, Chinese Academy of Medical Sciences & Peking Union Medical College, Beijing, China

^2^Laboratory of Endocrinology, Department of Endocrinology, National Health Commission, Peking Union Medical College Hospital, Chinese Academy of Medical Sciences & Peking Union Medical College, Beijing, China

*** Correspondence:**Quan Liao,
lqpumc@126.com

^#^Ya Hu and Xiang Zhang contributed equally to this work.

# Supplementary Figures

**
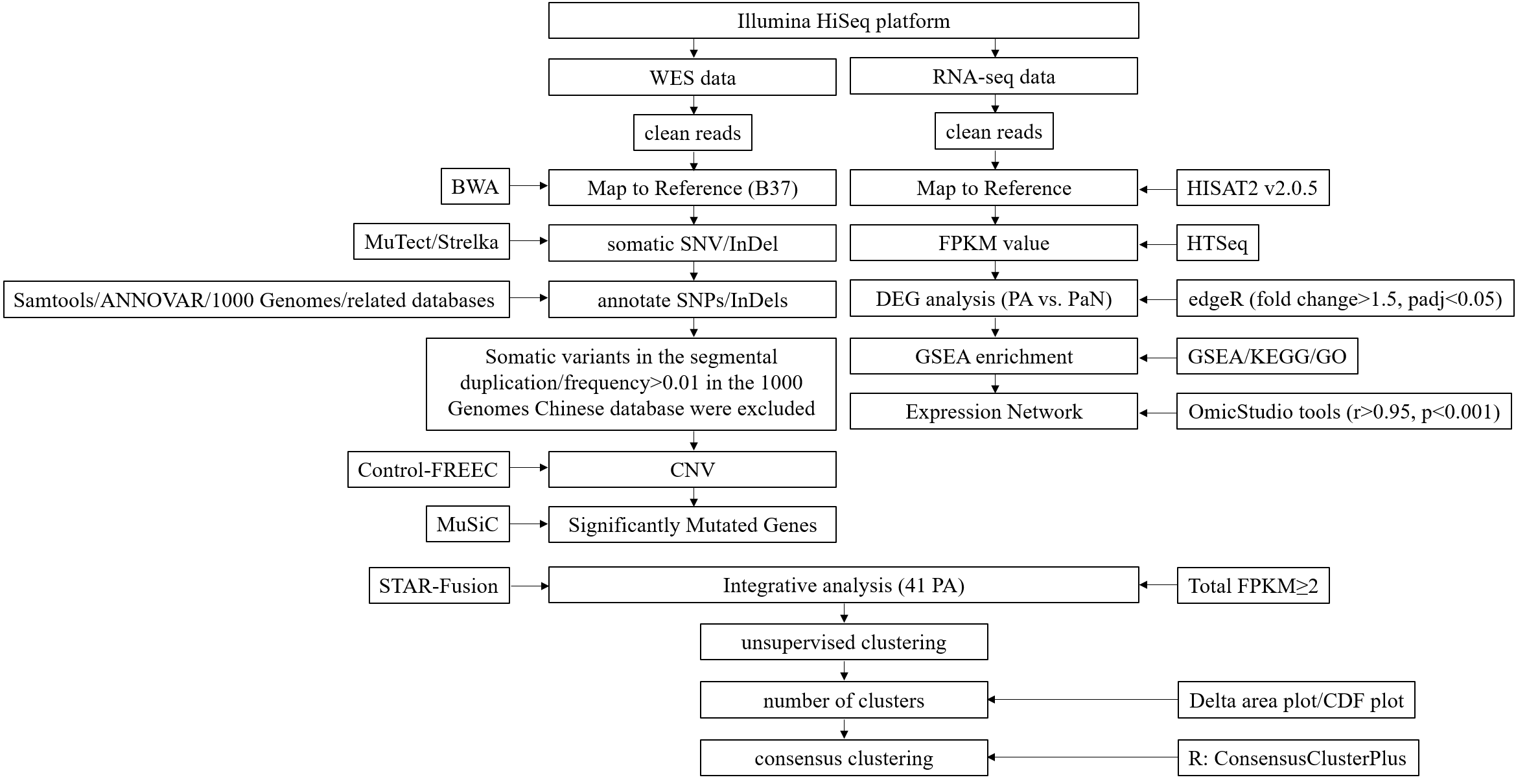
**

**Figure S1.** Flowchart of data analysis in the present study. PA, parathyroid adenoma; PaN, normal parathyroid; DEG, differentially expressed gene


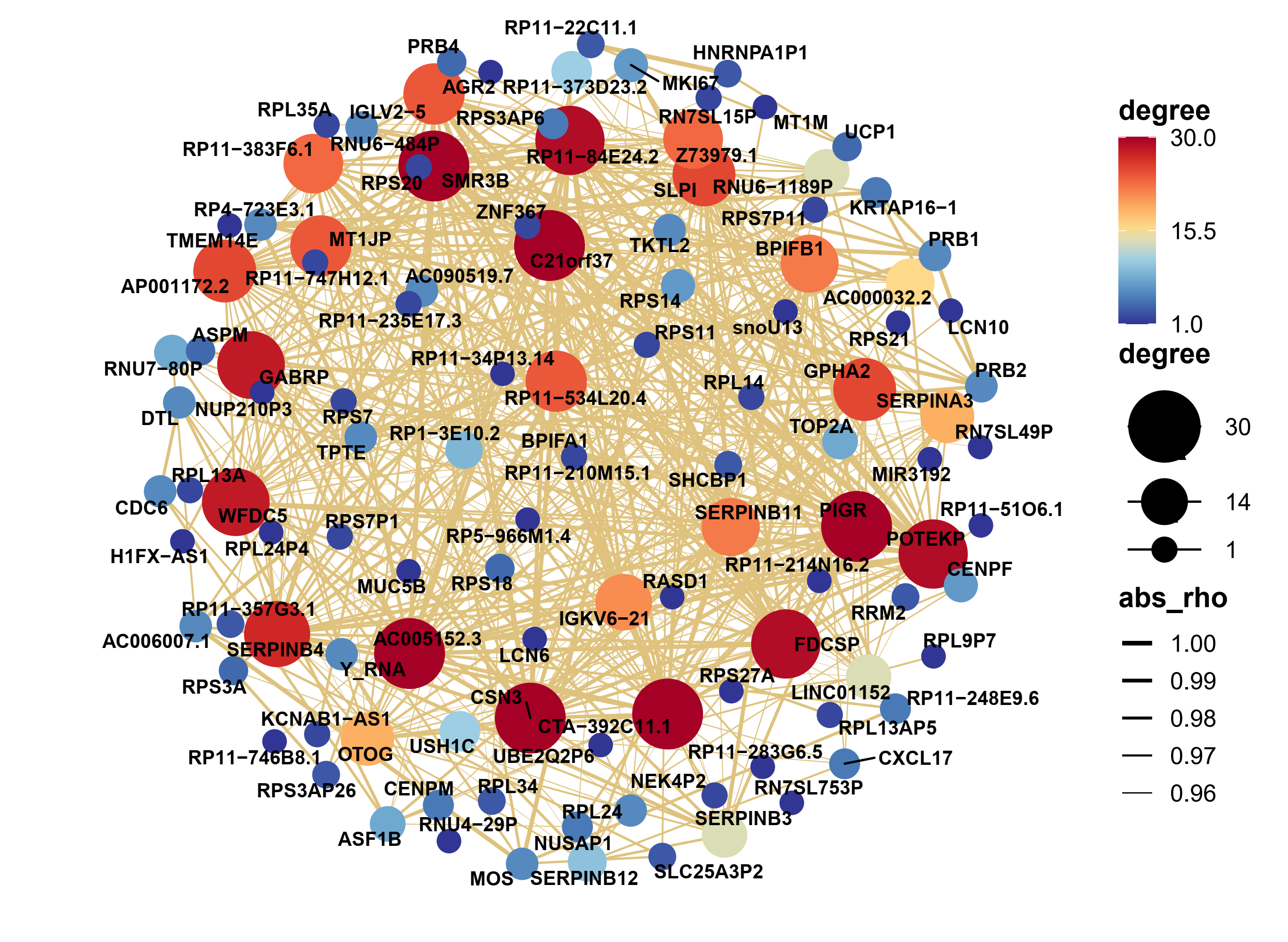


**Figure S2.** The expression network of the differentially expressed genes in parathyroid adenoma. Degree reflects the connections of the gene with others. Gene with high degree is shown as red and big circle. Gene with low degree was shown as blue and small circle. Abs_rho represents the Pearson correlation coefficient

**

**

**Figure S3.** Relative change in the area under the cumulative distribution function curve during the consensus clustering analysis of RNA-seq data showed that more than 10 clusters could not improve clustering performance
